# Suppressive effects of methylthiouracil on polyphosphate‐mediated vascular inflammatory responses

**DOI:** 10.1111/jcmm.12925

**Published:** 2016-07-15

**Authors:** Gahee Min, Sae‐Kwang Ku, Seongdo Jeong, Moon‐Chang Baek, Jong‐Sup Bae

**Affiliations:** ^1^ College of PharmacyCMRIResearch Institute of Pharmaceutical SciencesBK21 Plus KNU Multi‐Omics based Creative Drug Research TeamKyungpook National UniversityDaeguKorea; ^2^ Department of Anatomy and HistologyCollege of Korean MedicineDaegu Haany UniversityGyeongsanKorea; ^3^ Department of Molecular MedicineCMRISchool of MedicineKyungpook National UniversityDaeguKorea

**Keywords:** drug repositinging, methylthiouracil, polyphosphate, inflammation, barrier integrity

## Abstract

Drug repositioning is used to discover drug candidates to treat human diseases, through the application of drugs or compounds that are approved for the treatment of other diseases. This method can significantly reduce the time required and cost of discovering new drug candidates for human diseases. Previous studies have reported pro‐inflammatory responses of endothelial cells to the release of polyphosphate (PolyP). In this study, we examined the anti‐inflammatory responses and mechanisms of methylthiouracil (MTU), which is an antithyroid drug, and its effects on PolyP‐induced septic activities in human umbilical vein endothelial cells (HUVECs) and mice. The survival rates, septic biomarker levels, behaviour of human neutrophils and vascular permeability were determined in PolyP‐activated HUVECs and mice. MTU suppressed the PolyP‐mediated vascular barrier permeability, up‐regulation of inflammatory biomarkers, adhesion/migration of leucocytes, and activation and/or production of nuclear factor‐κB, tumour necrosis factor‐α and interleukin‐6. Furthermore, MTU demonstrated protective effects on PolyP‐mediated lethal death and the levels of the related septic biomarkers. Therefore, these results indicated the therapeutic potential of MTU on various systemic inflammatory diseases, such as sepsis or septic shock.

## Introduction

Finding new molecular targets or mechanistic pathways provides unique ways to translate research findings into new drugs. However, this requires excessive amount of money, effort, and time, primarily due to the step‐by‐step barriers involved in drug development processes 1. Drug repositioning is the application of compound or biological drugs that have been approved or that have failed to treat certain diseases, for the treatment of new diseases as long as the drugs are not toxic and are useful for curing other diseases 2, 3. Critical data on the approved drugs, such as their formulation, toxicity and pharmacology, are available because they have already been studied, attempted, and approved by the Food and Drug Administration (FDA) for the treatment of certain diseases. Therefore, these drugs can be immediately tested in clinical trials as novel drug candidates for diseases other than those approved by the FDA 2, 3.

Inorganic polyphosphate (PolyP), which is a linear polymer that is made up of several orthophosphate residues that are linked by adenosine triphosphate‐like phosphoanhydride bonds 4, is present in all bacterial and animal cells 5. Recent studies that were mostly conducted on microorganisms have reported a number of diverse biological functions of PolyP, including inflammation, apoptosis, proliferation and blood coagulation, in mammalian systems 6, 7, 8, 9. Our recent studies indicated pro‐inflammatory activities of PolyP, such as mediating vascular hyperpermeability, increasing the adhesion and migration of leucocytes, and up‐regulating the expression of cell adhesion molecules (CAMs), including vascular cell adhesion molecule‐1 (VCAM‐1), intercellular adhesion molecule‐1 (ICAM‐1) and E‐selectin 9, 10, 11.

In our research on the repositioning of 1,163 FDA‐approved drugs, 327 drugs that are related to vascular inflammation and infection were selected for repositioning. A high‐content screening system (Operetta, PerkinElmer, Inc., Waltham, MA, USA) was used to select the compounds that modulated the PolyP‐mediated disruption of the vascular endothelium, and we found that methylthiouracil (MTU), which is an antithyroid drug, had suppressive effects on the PolyP‐mediated septic responses. Based on our previous findings that demonstrated the potential effects of PolyP on vascular inflammatory responses 9, 10, 11, this study was conducted to investigate the mechanisms underlying the suppressive actions of MTU on PolyP‐induced septic responses in human endothelial cells and mice.

## Methods

### Reagents

**Figure 1:**
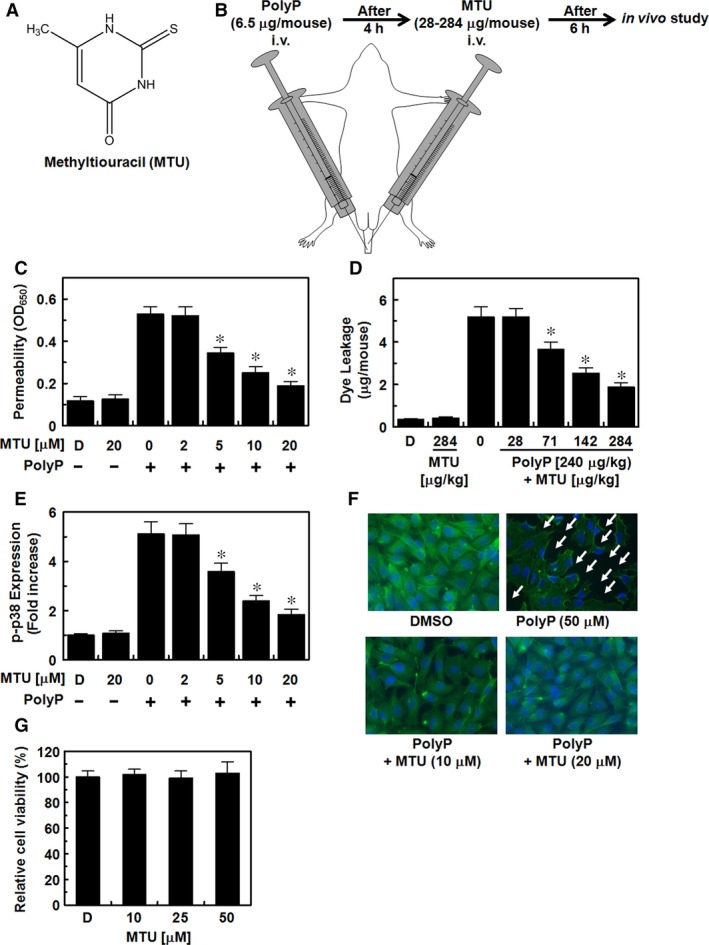
Effects of methylthiouracil (MTU) on Polyphosphate P (PolyP)‐induced barrier disruptions *in vitro* and *in vivo*. (**A**) The chemical structure of MTU. (**B**) An illustration of the protocol of the *in vivo* study. (**C**) The effects of various concentrations of MTU on PolyP‐induced (50 μM, 4 hrs) barrier disruption, which was monitored by the flux of Evans blue‐bound albumin across human umbilical vein endothelial cells (HUVECs). (**D**) The effects of MTU on PolyP‐induced [3.5 μg/mouse, intravenous (i.v.)] vascular permeability in mice were examined by the flux of Evans blue in the mice (expressed μg/mouse, *n* = 5). (**E**) After activation with PolyP (50 μM, 4 hrs), the HUVECs were treated with different concentrations of MTU for 6 hrs. The effects of each compound on the expression of PolyP‐mediated phosphorylated (phospho) p38 (p‐p38) were determined with an (ELISA). **(F)** Staining for F‐actin. The HUVEC monolayers grown on glass coverslips were stimulated with PolyP (50 μM, 4 hrs) and then treated with MTU for 6 hrs. F‐actin was examined with immunofluorescent staining. The arrows indicate intercellular gaps. (**G**) Effects of MTU (0–50 μM) on cellular viability. The results are expressed as means ± standard error of the mean (SEM) of three independent experiments. D indicates vehicle [0.5% dimethyl sulphoxide (DMSO)] only. *P < 0.05 *versus* PolyP (B–D).

MTU (Fig. 1A) was purchased from Abcam plc (Cambridge, MA, USA). Foetal bovine serum and Vybrant^®^ DiD were purchased from Thermo Fisher Scientific Inc. (Waltham, MA, USA). PolyP, Evans blue, crystal violet, 2‐mercaptoethanol, catalase‐polyethylene glycol and antibiotics (penicillin G and streptomycin) were purchased from Sigma‐Aldrich Co. LLC (St. Louis, MO, USA).

### Animals and husbandry

Male mice (C57BL/6 strain; average weight, 27 g; 6–7 weeks old) were obtained from Orient Bio Co., Ltd. (Sungnam, Korea) and used after a 12‐day acclimatization period. The mice were maintained as described previously 12, 13. Briefly, the animals were housed 5 per polycarbonate cage under controlled temperature (20–25°C) and humidity (40–45% RH) and a 12:12–hrs light/dark cycle. Animals received a normal rodent pellet diet and water *ad libitum* during the acclimatization. All of the animals were handled in accordance with the *Guidelines for the Care and Use of Laboratory Animals* that was issued by Kyungpook National University (IRB No. KNU 2016‐54).

### Cell culture

Primary human umbilical vein endothelial cells (HUVECs) were purchased from Cambrex Corporation (Charles City, IA, USA) and maintained as previously described 12, 13, 14, 15, 16. Briefly, the cells were cultured to confluency at 37°C and 5% CO_2_ in endothelial basal medium (EBM)‐2 basal media supplemented with growth supplements (Cambrex Bio Science, Charles City, IA, USA). The neutrophils were freshly isolated from whole blood (15 ml) that was obtained by the venipuncture of five healthy volunteers and maintained as previously described 17, 18.

### Permeability assay *in vitro*

To examine any changes in vascular permeability in response to increasing concentrations of MTU (0–20 μM), the flux of Evans blue‐bound albumin was measured, as described previously 19. Briefly, HUVECs were plated (5 × 10^4^/well) in 3‐μm pore size, 12‐mm diameter transwells for 3 days. Confluent HUVEC monolayers were treated with increasing concentrations of MTU (0–20 μM) for 6 hrs and then activated with PolyP (50 μM) for 4 hrs. Transwell inserts were then washed with PBS (pH 7.4) containing 0.5 ml of Evans blue (0.67 mg/ml) and suspended in growth medium containing 4% bovine serum albumin (BSA). Fresh growth medium was then added to the lower chamber, and the medium in the upper chamber was replaced with Evans blue/BSA. Ten minutes later, optical density was measured at 650 nm in the lower chamber.

### ELISA of phosphorylated (phospho) p‐38

A commercially available ELISA kit (Cell Signaling Technology, Inc., Danvers, MA, USA) was used to measure the expression levels of phospho p‐38.

### Immunofluorescence staining

Confluent HUVECs on glass coverslips that were coated with 0.05% Poly‐L‐Lysine were maintained for 2 days. The HUVECs were then activated with PolyP (50 μM, 4 hrs) with or without MTU (10 or 20 μM, 6 hrs). The cytoskeletal staining was assessed as previously described 20. Briefly, the cells were fixed in 4% formaldehyde in PBS (v/v) for 15 min. at room temperature, permeabilized in 0.05% Triton X‐100 in PBS for 15 min., and blocked in blocking buffer (5% BSA in PBS) overnight at 4°C. Then, the cells were incubated with F‐actin‐labelled fluorescein phalloidin (F 432; Molecular Probes, Invitrogen, Waltham, MA, USA), nuclei were counterstained with 4,6‐diamidino‐2‐phenylindole dihydrochloride (DAPI) and cells were visualized by confocal microscopy at a 630× magnification (TCS‐Sp5, Leica microsystem, Germany).

### Levels of expression of the protein and mRNA of CAMs

The confluent monolayers of the HUVECs were treated with PolyP (50 μM) for 16 hrs for VCAM‐1 and ICAM‐1 or 24 hrs for E‐selectin and then with MTU (10 or 20 μM) for 6 hrs. A whole‐cell ELISA was performed to determine the levels of expression of the ICAM‐1, E‐selectin and VCAM‐1 proteins on the HUVECs, as previously described 21, 22. Briefly, confluent monolayers of HUVECs were treated with MTU for 6 hrs after treatment of polyP (50 μM) for 16 hrs (VCAM‐1 and ICAM‐1) or 24 hrs (E‐selectin). The medium was removed, cells were washed with PBS, and fixed by adding 50 μl of 1% paraformaldehyde for 15 min. at room temperature. After washing, 100 μl of mouse anti‐human monoclonal VCAM‐1 (clone; 6C7.1), ICAM‐1 (clone; P2A4) and E‐selectin (clone; P2H3) antibodies (Millipore Corporation (Billerica, MA, USA), 1:50 each) were added.

For the real‐time polymerase chain reaction, RNA was isolated with TRI Reagent (Thermo Fisher Scientific Inc.) according to the manufacturer's protocol. The real‐time polymerase chain reaction was performed as previously described 11. Briefly, an aliquot (5 μg) of extract RNA was reverse transcribed into first‐strand cDNA with a PX2 Thermal Cycler (Thermo Scientific) using 200 U/μl M‐MLV reverse‐transcriptase (Invitrogen) and 0.5 mg/μl of oligo(dT)‐adapter primer (Invitrogen) in a 20‐μl reaction mixture. Real‐time PCR for VCAM‐1, ICAM‐1, E‐selectin and α‐actin was performed using a Mini Opticon Real‐Time PCR System (Bio‐Rad, Hercules, CA, USA) and iQ SYBR Green Supermix (Bio‐Rad).

### Cell–cell adhesion assay

The adhesion of purified human neutrophils to the HUVECs was tested by fluorescent labelling, as previously described 22. Briefly, after the neutrophils were labelled with fluorescein, confluent HUVECs were activated with PolyP (50 μM, 4 hrs) and then incubated with MTU (0–20 μM, 6 hrs). The percentage of adherent neutrophils was calculated as previously described 22. Briefly, the fluorescence of labelled cells was measured (total signal) using a fluorescence microplate reader (Tecan Austria GmbH, Austria). After incubation for 1 hr at 37°C, the non‐adherent cells were removed by washing four times with pre‐warmed RPMI, and the fluorescent signals of adherent cells were measured by previously described methods. The percentage of adherent neutrophils was calculated by the formula: % adherence = (adherent signal/total signal) × 100.

### *In vitro* migration assay

The migration of purified human neutrophils to the HUVECs was evaluated as previously described 23. MTU‐treated (0–20 μM, 6 hrs) confluent HUVECs were activated with PolyP (50 μM, 4 hrs). Purified human neutrophils were then applied to the upper chamber, and the migration index was measured as previously described 23. Briefly, transwell plates were then incubated at 37°C in 5% CO_2_ for 2 hrs. Cells in the upper chamber were then aspirated, followed by the removal of non‐migrating cells on top of the filter by using a cotton swab. Human neutrophils on the lower side of the filter were fixed with 8% glutaraldehyde and stained with 0.25% crystal violet in 20% methanol (w/v). Experiments were repeated twice per well in duplicate wells, and nine randomly selected high power microscopic fields (HPF, 200×) were counted. The results are presented as Migration Indices.

### *In vivo* permeability and leucocyte migration assay

The mice were treated with PolyP (6.5 μg/mouse, intravenous administration) or 0.5% dimethyl sulphoxide (DMSO), which was used as a control (Fig. 1B). The mice were then intravenously administered MTU (28–284 μg/kg), and 1% Evans blue dye solution in normal saline was injected after 4 hrs. The vascular permeability and leucocyte migration were determined as previously described 24, 25. For vascular permeability, 6 hrs later, the mice were sacrificed and peritoneal exudates were collected by washing cavities with 5 ml of normal saline and by centrifuging at 200 *g* for 10 min. The absorbance of the supernatant was read at 650 nm. Vascular permeabilities are expressed as μg of dye/mouse that leaked into the peritoneal cavity, and were determined using a standard curve. For leucocyte migration, after the mice were killed, peritoneal cavities were washed with 5 ml of normal saline. Obtained samples (20 μl) of the peritoneal fluid were mixed with 0.38 ml of Turk's solution (0.01% crystal violet in 3% acetic acid), and number of leucocytes were counted using a light microscope.

### ELISA for nuclear factor (NF)‐κB, extracellular signal‐regulated kinase (ERK)1/2, interleukin (IL)‐6 and tumour necrosis factor (TNF)‐α

Commercially available ELISA kits were used to determine the levels of expression of total and phospho‐NF‐κB p65 (# 7174 and # 7173, Cell Signaling Technology, Inc.) and total/phospho ERK1/2 (R&D Systems, Inc., Minneapolis, MN, USA) in the nuclear lysates of HUVECs and the levels of IL‐6 and TNF‐α (R&D Systems, Inc.) in the cell culture supernatants of the HUVECs.

### PolyP‐induced lethal model

PolyP (6.5 μg/mouse) in DMSO or 0.5% DMSO, which was a control, was intravenously injected into the mice. At 12 hrs or 50 hrs after the PolyP injection, male C57BL/6 mice were administered MTU (142 or 284 μg/kg). Animal survival was monitored every 6 hrs after the PolyP65 injections for 132 hrs.

### Measurements of organ injury markers

The plasma levels of aspartate transaminase, alanine transaminase, blood urea nitrogen and creatinine were measured with commercial assay kits (Pointe Scientific, Inc., Canton, MI, USA).

### Statistical analysis

Each experiment was independently performed at least three times, and each value was expressed as the mean ± standard error of mean. SPSS for Windows (version 16.0; IBM Corporation, Armonk, NY, USA) was used to evaluate the statistical significance of the differences between the test groups. Statistical relevance was determined with one‐way anova and/or Tukey's *post hoc* test. A Kaplan–Meier analysis was used to evaluate the survival data on the outcome of the caecal ligation and puncture‐induced sepsis. *P* < 0.05 were considered statistically significant.

## Results and discussion

### Inhibitory effects of MTU on the PolyP‐induced hyperpermeability

Vascular permeability was assessed to test the effects of MTU (Fig. 1A) on the PolyP‐induced disruptions of the vascular barrier as the endothelial barrier integrity is cleaved by PolyP 9, 10. Our previous studies reported the PolyP parameters (50 μM and 4 hrs) that optimize the disruption of endothelial integrity 9, 10. The cells were activated with PolyP (50 μM) for 4 hrs and then various concentrations of MTU for 6 hrs. The results showed the inhibitory effects of MTU on the PolyP‐mediated hyperpermeability, with the optimal dose occurring at concentrations above 5 μM (Fig. 1C). Furthermore, MTU alone (20 μM) did not alter the barrier integrity of the HUVECs (Fig. 1C). Next, MTU was intravenously injected into mice with PolyP‐mediated hyperpermeability. The results showed that PolyP enhanced vascular permeability, and this was suppressed by MTU (Fig. 1D). As the average blood volume is 72 ml/kg 26 and the average weight of the mice that were used in this study was 27 g, the concentrations of MTU (28, 71, 142 and 284 μg/kg) that were administered were equal to 2, 5, 10 and 20 μM, respectively, in the blood.

Vascular inflammatory inducers, such as lipopolysaccharide and high‐mobility group box 1 protein, mediate inflammatory responses by activating p38 mitogen‐activated protein kinase (MAPK) 27, 28. Therefore, the effects of MTU on the PolyP‐induced activation of p38 MAPK were determined. The results revealed that MTU inhibited the PolyP‐induced up‐regulation of phospho p38 expression (Fig. 1E).

Previous studies have indicated the importance of cytoskeletal proteins for maintaining cell integrity and shape 29 and the involvement of vascular integrity in the detachment of cell–cell contact and redistribution of the actin cytoskeleton 30, 31. Thus, the effects of MTU on PolyP‐mediated actin cytoskeletal arrangement in HUVECs were examined by staining the HUVECs with fluorescein phalloidin‐labelled F‐actin. Compared to the control HUVECs that displayed an irregular distribution of F‐actin, the disruption in the barrier integrity that was induced by treatment with PolyP (50 μM) was demonstrated by the formation of paracellular gaps in the HUVECs, and these were reduced by treatment with MTU (20 μM) (Fig. 1F). To exclude the possibility that the barrier‐protecting effects of MTU were because of the cellular cytotoxicity of MTU, cellular viability assays were conducted. As shown in Figure 1G, MTU was not cytotoxic in the HUVECs at concentrations up to 50 μM.

Because the high morbidity and mortality that are seen in patients with serious inflammatory diseases result from the disruption of vascular integrity 32 and because the reagents used to treat a number of inflammatory diseases are designed to inhibit vascular hyperpermeability 33, our results indicated the potential of MTU as a therapeutic agent in various vascular inflammatory diseases.

### Effects of MTU on the PolyP effects on CAM expression and neutrophil adhesion and migration

**Figure 2:**
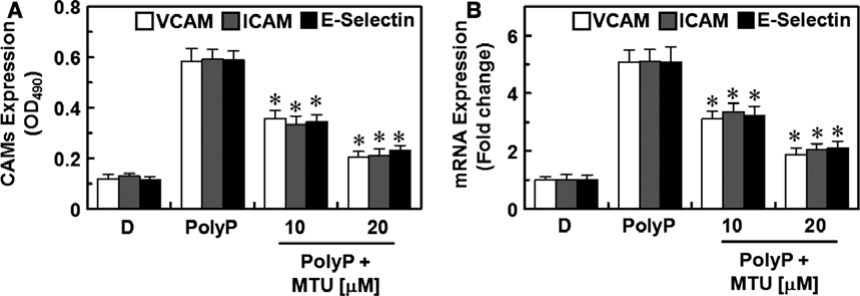
Effects of methylthiouracil (MTU) on the PolyP‐induced expression of cellular adhesion molecules (CAMs) in human umbilical vein endothelial cells (HUVECs). (**A**) The PolyP‐mediated (50 μM) levels of expression of vascular cell adhesion molecule‐1 (VCAM‐1; white bar), intercellular adhesion molecule‐1 (ICAM‐1; grey bar), and E‐selectin (black bar) proteins in HUVECs were analysed with whole‐cell ELISA after treating the monolayers with MTU. (**B**) The PolyP‐mediated (50 μM) levels of expression of VCAM‐1 (white bar), ICAM‐1 (grey bar), and E‐selectin (black bar) transcription (mRNA) in HUVECs were analysed with whole‐cell ELISA after treating the monolayers with MTU. The results are expressed as the means ± SEM of three independent experiments. *P < 0.05 *versus* PolyP.

**Figure 3:**
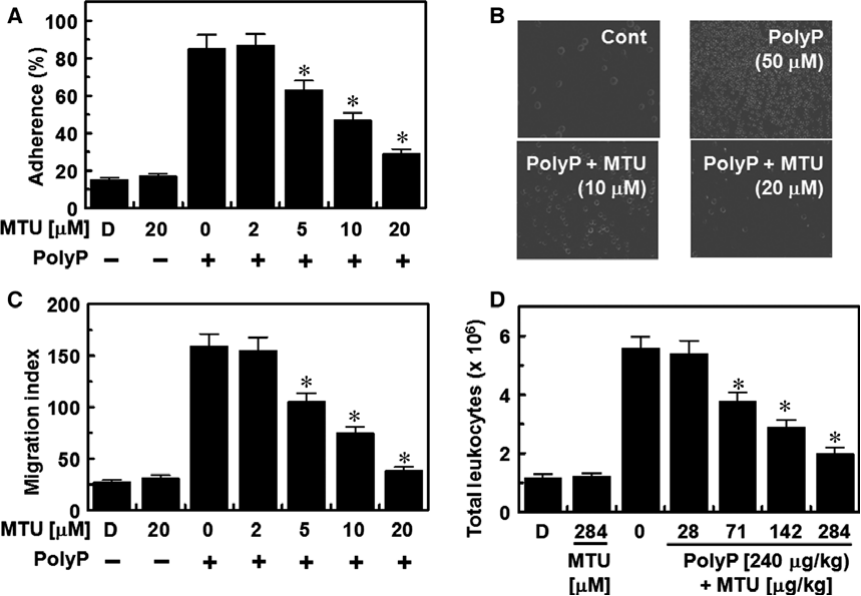
Effects of methylthiouracil (MTU) on PolyP‐induced cell adhesion and migration of neutrophils. (**A**) The PolyP‐mediated (50 μM) adherence of neutrophils to human umbilical vein endothelial cells (HUVECs) was analysed after treating the cells with MTU. (**B**) Representative photomicrographs of neutrophil adhesion to HUVECs. Cont indicates control. (**C**) The PolyP‐mediated (50 μM) migration of neutrophils through HUVEC monolayers was analysed after treating the cells with MTU. (**D**) Effects of MTU on PolyP‐induced (3.5 μg/mouse, i.v.) leucocyte migration in mice (expressed as ×10^6^, *n* = 5). The results are expressed as the means ± SEM of three independent experiments. D indicates vehicle [0.5% dimethyl sulphoxide (DMSO)] only. *P < 0.05 *versus* PolyP.

Previous studies have confirmed the pivotal roles of CAMs (VCAM‐1, ICAM‐1 and E‐selectin) during cell–cell adhesion in processes of vascular inflammation 34, 35. Thus, inhibiting the expression of CAMs is considered a promising therapeutic approach for treating vascular inflammatory diseases. We recently showed that the levels of expression of CAMs are increased by PolyP, thereby stimulating leucocyte behaviours, such as adhesion and migration, towards HUVECs 9, 10. In this study, we determined the effects of MTU on the levels of expression of CAMs and on the adhesion and migration of leucocytes towards HUVECs, which were both affected by PolyP. The results showed that MTU suppressed the increases in the levels of protein and transcript expression of CAMs (Fig. 2A and B). In addition, the enhancement in the expression of CAMs correlated with the increased binding and migration of leucocytes to PolyP‐treated HUVECs, and this was inhibited by MTU treatment in a concentration‐dependent manner (Fig. 3A–C). To confirm these results *in vivo*, we examined the effects of injected MTU on PolyP‐induced leucocyte migration in mice. PolyP increased the number of migrated leucocytes in the peritoneal cavities of mice, and this was reduced by MTU treatment (Fig. 3D). Collectively, the results of this study showed that MTU downregulated PolyP‐mediated vascular inflammatory responses by inhibiting the augmentation by PolyP of inflammatory signalling pathways, such as the adhesion and migration of leucocytes to inflamed endothelium.

### Effects of MTU on the PolyP‐stimulated activation of NF‐κB/ERK and the production of IL‐6/TNF‐α

**Figure 4:**
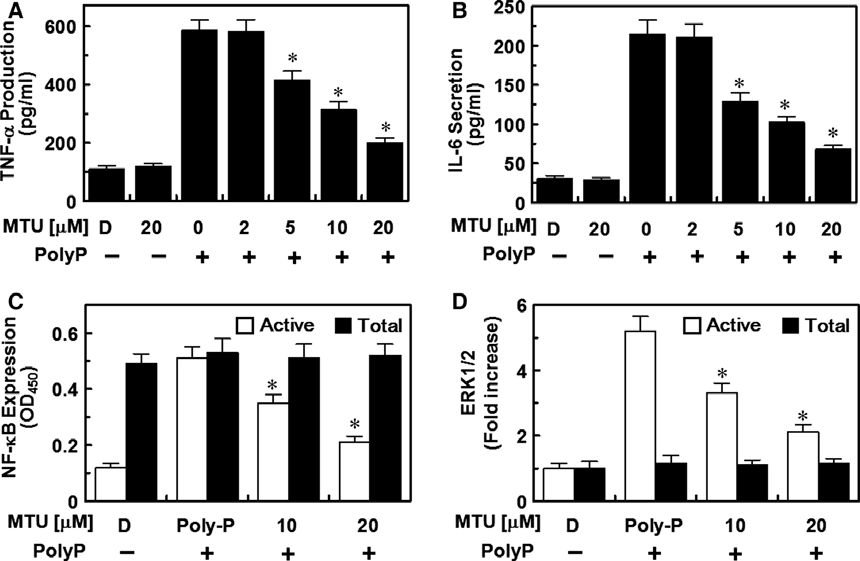
Effects of methylthiouracil (MTU) on the PolyP‐stimulated activation of nuclear factor (NF)‐κB/extracellular signal‐regulated kinase (ERK) 1/2 and production of interleukin (IL‐6)/tumour necrosis factor (TNF)‐α. The PolyP‐mediated (50 μM) production of TNF‐α (**A**) and IL‐6 (**B**) in human umbilical vein endothelial cells (HUVECs) was analysed after the treatment of the cells with the indicated concentrations of MTU for 6 hrs. (**C**) The PolyP‐mediated (50 μM) activation of phospho‐NF‐κB p65 (white bar) and total NF‐κB p65 (black bar) in the HUVECs was analysed after the treatment of the cells with MTU for 6 hrs. (**D**) The PolyP‐mediated (50 μM) activation of phospho‐ERK1/2 (white bar) and total ERK1/2 (black bar) in the HUVECs was analysed after the treatment of the cells with MTU for 6 hrs. D indicates vehicle [0.5% dimethyl sulphoxide (DMSO)] only. *P < 0.05 *versus* PolyP only.

On the basis of the results of this study and findings that the activation of NF‐κB or ERK and the increased production of TNF‐α or IL‐6 amplifies vascular inflammatory responses 36, 37, 38, 39, 40, we have been suggested that the activation and expression of these pro‐inflammatory molecules were suppressed by MTU. To confirm this hypothesis, the levels of activation and expression of these pro‐inflammatory molecules were measured with ELISA in PolyP‐activated and MTU‐treated HUVECs. The results showed that the increased levels of protein expression of TNF‐α and IL‐6 (Fig. 4A and B) and the increased activation of NF‐κB and ERK1/2 (Fig. 4C and D) that were induced by PolyP were reduced by MTU. Therefore, these results suggested that MTU can control important vascular inflammatory signalling pathways by regulating the molecules involved.

### Vascular protective effects of MTU in the PolyP‐induced lethality model

**Figure 5:**
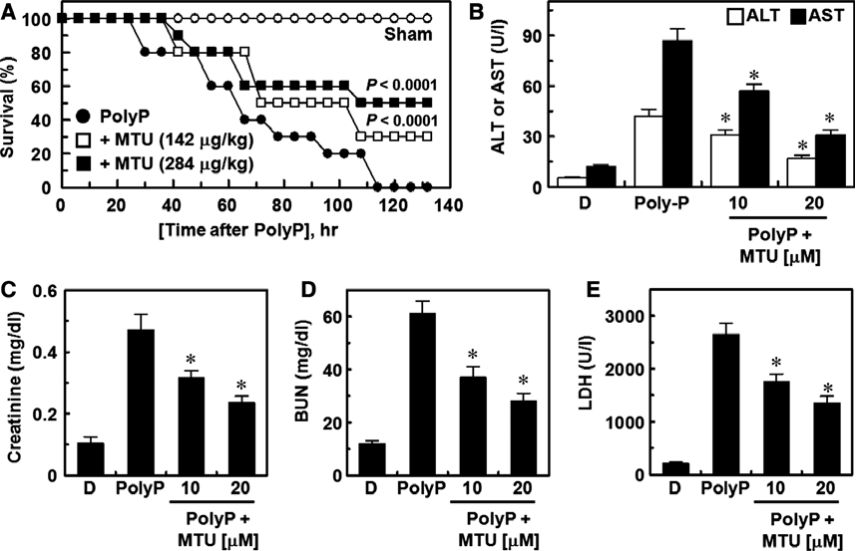
Effects of methylthiouracil (MTU) on PolyP‐induced lethality and organ damage markers. (**A**) Male C57BL/6 mice (*n* = 20) were intravenously administered MTU (142 μg/kg, □) or MTU (284 μg/kg, ■) 12 hrs and 50 hrs after PolyP (3.5 μg/mouse, i.v.) injections. Animal survival was monitored every 6 hrs after the PolyP injections for 132 hrs. The PolyP‐injected mice (●) and control mice (○) were administered 0.5% dimethyl sulphoxide (DMSO) (*n* = 20). A Kaplan–Meier survival analysis was used to determine the overall survival rates *versus* the PolyP‐treated mice. The plasma levels of the hepatic injury markers aspartate transaminase (AST) and alanine transaminase (ALT) (**B**), renal injury markers creatinine (**C**) and bun urea nitrogen (BUN) (**D**), and tissue injury marker lactate dehydrogenase (LDH) (**E**) were measured (*n* = 5) 72 hrs after the PolyP injections. D indicates vehicle (0.5% DMSO) only. *P < 0.05 *versus* PolyP alone.

Finally, we have been suggested that MTU would prevent PolyP‐mediated lethality in mice. To confirm this, mice were administered with MTU after PolyP injections. The results showed that treatment with a single dose of MTU (142 or 284 μg/kg, 12 hrs after the PolyP injection) did not prevent PolyP‐induced death (data not shown). Thus, MTU was administered twice (once at 12 hrs and then 50 hrs after the PolyP injection), which resulted in an increase in the survival rate from 0 to 50% in the Kaplan**–**Meier survival analysis (Fig. 5A, *P* < 0.0001). As the liver and kidney are major target organs of systemic inflammatory diseases and multiple organ failure is caused by systemic inflammatory diseases, such as sepsis and septic shock 41, we examined the plasma levels of tissue damage markers. As shown in Figure 5B–E, MTU reduced the polyP‐induced increases in the plasma levels of alanine transaminase and aspartate transaminase (markers of hepatic injury, Fig. 5B) and creatinine and blood urea nitrogen (markers of renal injury, Fig. 5C and D). In addition, the levels of lactate dehydrogenase, which is a marker of tissue injury, were reduced by MTU in PolyP‐injected mice (Fig. 5E).

The molecular mechanisms underlying the anti‐inflammatory effects of MTU on PolyP‐mediated septic responses may be mediated by the suppression of PolyP‐mediated hyperpermeability (Fig. 1C and D) through the inhibition of the activation of p38 (Fig. 1E). Furthermore, the inhibitory mechanisms of MTU on the interaction of leucocytes with endothelial cells are mediated by the inhibition of the expression of CAMs, such as VCAM, ICAM and E‐selectin (Figs 2 and 3). The underlying mechanisms of these anti‐inflammatory effects of MTU involve the down‐regulation of the production of inflammatory cytokines (TNF‐α and IL‐6, Fig. 4A and B) and the activation of inflammatory transcriptional factors (NF‐κB and ERK1/2, Fig. 4C and D).

Finally, the results of this study showed the protective activities of MTU on the vascular barrier disruptions induced by PolyP in both human endothelial cells and mice. Therefore, these results suggested that MTU is a potential candidate in the treatment of severe vascular inflammatory diseases.

## Conflict of interest

The authors declare no conflicts of interest.
